# Comparison of Postorthodontic Occlusal Force Distributions between Nonextraction and Extraction Treatment Approaches of Premolars for Orthodontic Treatment by Using T-Scan III System

**DOI:** 10.1055/s-0044-1789002

**Published:** 2024-11-07

**Authors:** Ratchawan Tansalarak, Nattapat Khamnuengsitthi, Mayurach Pipatphatsakorn

**Affiliations:** 1 Division of Orthodontics, Department of Preventive Dentistry, Faculty of Dentistry, Naresuan University, Phitsanulok, Thailand; 2 Division of Operative Dentistry, Department of Restorative Dentistry, Faculty of Dentistry, Naresuan University, Phitsanulok, Thailand

**Keywords:** bite force, T-Scan III, tooth loss, postorthodontic, dental extraction

## Abstract

**Objective** To compare bite force distributions in corresponding mouth regions between postorthodontic patients who underwent nonextraction and extraction approaches using the T-Scan III system (Tekscan, Inc, Massachusetts, United States).

**Materials and Methods** Thirty-six postorthodontic patients were divided into two groups: (1) 18 subjects with the nonextraction treatment and (2) 18 subjects with the extraction of four first-premolar treatments. The measurements were performed using the T-Scan to collect the occlusal bite force at the maximal intercuspal position to generate the bite force in the anterior region (incisors and canines) and posterior region (premolars and molars), the bite force in individual teeth, and the anteroposterior bite force ratio (A-P ratio).

**Statistical Analysis** The mean bite force in each region, individual teeth, and the A-P ratio were compared between the two groups by the Mann–Whitney's *U* -tests. Within-group comparisons of the mean bite force in each region were performed using Wilcoxon's signed rank tests.

**Results** The bite force in anterior and posterior regions, and the A-P ratio of the nonextraction group show no significant difference compared with the extraction group ( *p* > 0.05). For individual teeth (central incisors, lateral incisors, canines, second premolars, first molars, and second molars), each tooth exhibited nonsignificantly different bite forces ( *p* > 0.05) except for the second molars. The second molars in the nonextraction group had significantly less bite force than in the extraction group ( *p* = 0.001). The comparison of occlusal bite force between the right and left sides showed that in the nonextraction group, the right side had significantly greater force ( *p* < 0.05). In the extraction group, there was no significant difference between the sides ( *p* > 0.05).

**Conclusion** Both nonextraction and extraction orthodontic treated patients exhibited the similar bite force distribution patterns in regions and individual teeth except for the higher occlusal force on the second molars in the extractions compared with the nonextractions.

## Introduction

The masticatory performance has been reported to correlate with the occlusal bite force. 1 2 3 4 Many factors including gender, age, craniofacial morphology, malocclusion, occlusal contact area, number of teeth, body mass index, and regions of the dental arch in the oral cavity were associated with the amount of occlusal bite force. 4 5 6 7 8 9 10 11 Examining the relationship between bite force and masticatory performance in individuals with the previously mentioned oral conditions can provide insights into how these conditions affect bite force and mastication.

Previous investigators have evaluated the association between the number of teeth present and bite force. 3 8 9 12 13 14 They found that as the number of teeth decreased, both the occlusal bite force and the occlusal contact area decreased, while occlusal pressure increased. Dentate patients exhibited similar patterns, with a reduction in occlusal bite force due to a reduced number of residual teeth in the mouth. 12

A decrease in teeth in mouths was also found in orthodontic patients. Dental extractions or space-closure therapy were required to correct some types of malocclusions, such as crowding and missing teeth, resulting in fewer teeth in the final occlusion. 15 The occlusal force distribution in the normal occlusion achieved after the orthodontic treatment was expected to be that the posterior regions had a heavier occlusal bite force than that in the anterior regions to protect the anterior teeth while in the maximal intercuspal position (MIP). 16 17 In the same way, postorthodontic subjects had more bite force percentage on posterior teeth, especially in molar regions, and less bite force percentage on anterior teeth than nonorthodontic subjects. 18 19 However, Thabet et al in 2023 found occlusal force imbalances and high-loading bite force on molar regions in postorthodontic patients treated with premolars extraction. 19 Thus, at present, the doubt of whether the bite force distribution in postorthodontic patients with the extraction approach is different from the postorthodontic patients with nonextraction approaches (full dentition) still needs to be clarified.

According to the bite force measurement, previous studies evaluated the occlusal bite force by utilizing various occlusal digital indicators. 5 Thus, the size and thickness of the occlusal indicators varied by different manufacturers. 4 10 12 13 20 The thinner the occlusal indicators were, the more likely the measurement of the occlusal bite force could represent the normal function. 5 Despite its thinness, nondigital occlusal indicators such as shim-stock foils and articulating papers could provide only indirect occlusal forces, including mark spots on teeth and subjective feeling feedback. 21 22 23 In digitalized occlusal parameters, the T-Scan system (Tekscan, Inc, Massachusetts, United States) was a whole-arch occlusal record with a 100-µm thickness sensor. This simple and reproducible method could illustrate either the relative occlusal bite force (in percentage) of each tooth or each region of the mouth with real-time illustration and record on a connected computer. 18 24 The advantage of the percentage bite force was that it had more consistency than the absolute bite force when repeating the measurement. 24

Hence, the objective of this study was to compare the occlusal bite force distributed in each area of the mouth in postorthodontic patients between the nonextraction and extraction approaches by using the T-Scan system.

## Materials and Methods

This cross-sectional study was conducted after the approval of the Naresuan University Ethical Committee (IRB No. P100116/63) from September 2020 to February 2022. During the period, all finished orthodontic patients at the Orthodontic Clinic of Naresuan University Dental Hospital, Thailand, were examined and selected as participants based on the following: (1) completion of fixed edgewise orthodontic treatment and starting the retention phase within 1 year; (2) no edentulous space; (3) 24 teeth, excluding first premolars in extraction patients; and (4) 28 teeth, excluding third molars in nonextraction patients. Exclusion criteria included: (1) use of a removable denture; (2) orofacial pain during mandibular movement; (3) presence of joint sound; (4) unstable biting; and (5) any disorder associated with facial muscle weakness. Participants were divided equally into two groups based on the extraction approach: (1) subjects treated with the nonextraction approach (nonextraction group) and (2) subjects treated with four first-premolar extractions (extraction group). Orthodontic treatments for all participants were completed using conventional nondigital force measurements during the finishing and detailing phases. All participants were informed about the study and provided their informed consent before participation.

The same operator provided the T-Scan record with the subject sitting upright and the Frankfort plane parallel to the floor. The subject bit on the T-Scan sensor for 5 seconds at centric occlusion to collect occlusal bite force at the MIP. This procedure was performed during an appointment visit from 9 a.m. to 4 p.m. The recording procedure was repeated three times during the same visit, with a 5-minute resting interval between each recording. A large T-Scan HD sensor was used, and each sensor was applied to record data from only one participant.

**Fig. 1:**
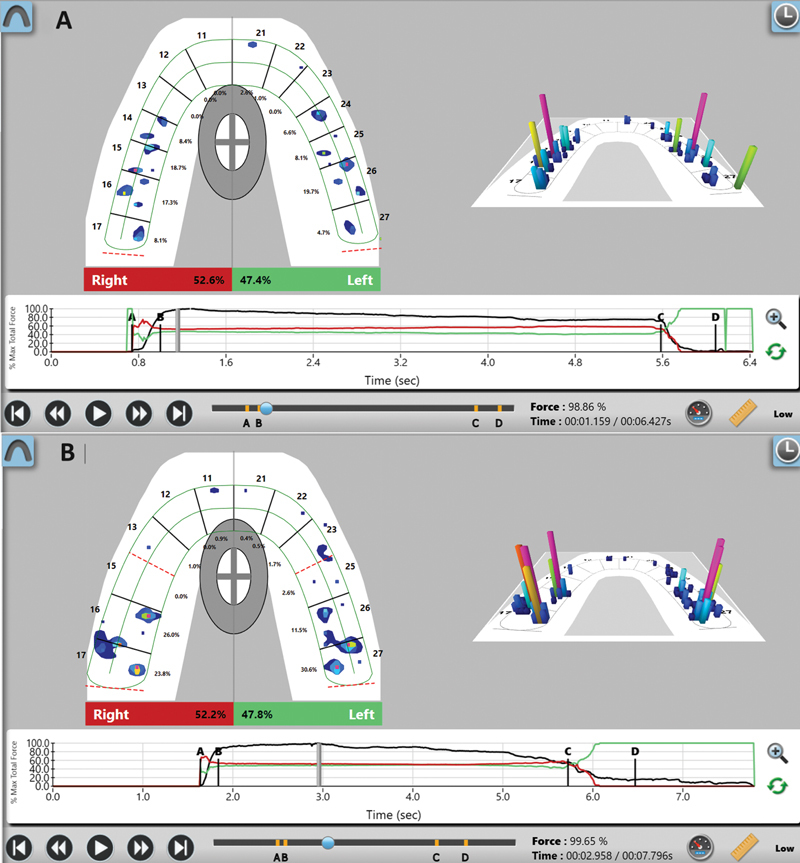
T-Scan recording windows of subjects treated by the nonextraction ( **A** ) and extraction ( **B** ). Both show the occlusal bite force in “maximum bite force” duration both in percentage of each tooth in the upper arch (left) and in three-dimensional bar chart (right).

In the T-Scan software, the two-dimensional and three-dimensional force view windows displayed each tooth's relative occlusal bite force selected using the “maximum intercuspation” command ( Fig. 1 ). In the software window, no first premolar was present in the subjects who underwent the extraction approach ( Fig. 1A ) compared with those in the nonextraction approach ( Fig. 1B ). The teeth were divided into two regions: (1) the anterior region, which included all incisors and canines and (2) the posterior region, which included all premolars and molars. Summations of the occlusal bite force of all teeth in each region were done. The three values of occlusal bite force from the repeated bites were averaged for each tooth and region. Each subject's anteroposterior bite force ratio (A-P ratio) was calculated by dividing the mean bite force of the anterior region by the mean bite force of the posterior region.

The occlusal bite force of each region was compared within-group by Wilcoxon's signed ranks test and between-group by Mann–Whitney's *U* -test. The bite force of each tooth was compared between the extraction and nonextraction groups by the independent sample *T* -test, and the one-way analysis of variance with a post hoc test. The confidence interval was set at 95%.

By comparing the occlusal forces of teeth obtained by the operator of this study with those from the expert, an interexaminer calibration was done within five subjects not included in this investigation. In addition, an intraexaminer calibration was done by which the operator of this study collected the occlusal force of teeth two times, of which the second collection had a 4-week interval from the first collection. The occlusal bite force of each tooth between the two sets was compared. The excellent correlation of interexaminer and intraexaminer calibration was achieved by the intraclass correlation coefficient (0.974 and 0.924, respectively). 25

## Results

In the study, a total of 36 participants were enrolled and categorized into two distinct groups: (1) the nonextraction group consisted of 18 subjects and (2) the extraction group also comprised 18 subjects. Both groups showed similar subjects characteristics ( *p* < 0.05) except for gender ( *p* = 0.048) ( Table 1 ). The nonextraction group mainly consisted of males (66.7%), while the extraction group was females in the majority (66.7%). The skeletal classification in the nonextraction group was mostly in Class I and Class III skeletal classification, while the extraction group predominated with Class I and Class II skeletal classification. Both groups showed similar pretreatment dental malocclusions, with the majority of Class I malocclusions followed by Class III and Class II division 1 malocclusions. All subjects' mean ages were 23.6 ± 6.5 years without a significant difference between the two groups ( *p* = 0.203). Additionally, the subjects' characteristics are described in Table 1 , which includes gender, types of pretreatment malocclusions, skeletal characteristics, and types of orthodontic treatment. The occlusion was assumed to be normal since all subjects had completed orthodontic treatment.

**Table 1:** Comparison of demographic subjects' characteristics between the nonextraction and extraction groups ( *n* = 18 for each group)

Subjects' characteristic	Treatment		*p* -Value
	Nonextraction, *n* (%)	Extraction, *n* (%)	
Gender			
Male	12 (66.7)	6 (33.3)	0.048 a
Female	6 (33.3)	12 (66.7)	
Skeletal classification			
Class I	8 (44.4)	8 (44.4)	0.080
Class II	2 (11.1)	7 (38.9)	
Class III	8 (44.4)	3 (16.7)	
Pretreatment dental classification			
Class I	9 (50.0)	8 (44.4)	0.084
Class II	1 (5.6)	6 (33.3)	
Class III	8 (44.4)	4 (22.2)	
Types of orthodontic treatment			
Corrective treatment	14 (77.8)	16 (88.8)	0.670
Two-phase treatment	2 (11.1)	1 (5.6)	
Combined surgery	2 (11.1)	1 (5.6)	

a Statistically significant at *p* < 0.05 by chi-square tests.

Both groups had bite force distributions in the posterior region significantly higher than in the anterior region ( *p* < 0.001) ( Table 2 ). Compared between the groups, the bite force of the anterior region, the posterior region, and the A-P ratio of the nonextraction group had no significant difference from those of the extraction group (anterior: *p* = 0.481; posterior: *p* = 0.443; A-P ratio: *p* = 0.668).

**Table 2:** Comparison of occlusal bite forces in anterior and posterior regions of postorthodontic subjects with extraction and nonextraction approaches ( *n* = 18 for each group)

Treatment	Age (y ± SD)	Bite force (%)				*p* -Value	Anterior–posterior bite force ratio	
		Anterior region		Posterior region				
		± SD	95% CI	± SD	95% CI		± SD	95% CI
Nonextraction	22.5 ± 5.7	15.87 ± 13.31	9.25–22.49	83.43 ± 13.34	76.80–90.07	<0.001 a	0.18 ± 0.18	0.09–0.28
Extraction	24.6 ± 7.2	12.09 ± 8.84	7.69–16.49	87.88 ± 8.63	83.59–92.18	<0.001 a	0.15 ± 0.12	0.09–0.20
*p* -Value	0.203	0.481		0.443			0.668	

Abbreviations: CI, confidence interval; SD, standard deviation.

a Statistically significant at *p* < 0.01 by Wilcoxon's signed ranks test for within-group comparison and Mann–Whitney's test for between-group comparison.

According to the bite force distribution on each type of tooth ( Table 3 ), the first premolars were omitted due to the removals of the teeth in the extraction group. Both groups showed the lowest bite force on lateral incisors and the highest on the first and second molars. By comparing the extraction and nonextraction groups, the tooth with the same type except for the second molars exhibited no significantly different bite forces ( *p* > 0.05). The second molars of the extraction group had a higher bite force than those of the nonextraction group ( *p* = 0.001).

**Table 3:** Comparison of occlusal bite forces of each tooth of postorthodontic treatment subjects with extraction and nonextraction approaches

Tooth Treatment	Individual teeth bite force distribution (% ± SD)						
	Central incisor	Lateral incisor	Canine	First premolar	Second premolar	First molar	Second molar
Nonextraction	3.11 ± 4.27 a b	1.03 ± 1.48 a	3.26 ± 3.16 a b	6.68 ± 5.18 b	6.60 ± 4.34 b	14.51 ± 8.17 ^c^	13.43 ± 8.92 ^c^
Extraction	1.37 ± 1.55 a	1.10 ± 1.77 a	3.58 ± 3.94 a b	–	6.25 ± 4.34 b	16.68 ± 7.23 ^c,d^	20.87 ± 9.37 ^d^
*p* -Value	0.173	0.950	0.830	–	0.744	0.220	0.001 b

Abbreviation: SD, standard deviation.

Notes: Each tooth type contained right and left sides ( *n* = 36 for each tooth in each group except for the first premolar in the extraction group ( *n* = 0). Values with the same uppercase letter indicate no significant difference among individual teeth of tooth treatment by one-way analysis of variance with post hoc analysis.

a Statistically significant at *p* < 0.05.

b Statistically significant at *p* < 0.01 by the Mann–Whitney's *U* -test for between-group comparison.

The comparison of the occlusal force balance between the right and left sides was conducted for both the nonextraction and extraction groups ( Table 4 ), indicating that in the nonextraction group, the occlusal bite force on the right side was significantly greater than on the left side ( *p* = 0.014). In contrast, the comparison in the extraction group revealed no significant difference between the right and left posterior forces ( *p* > 0.05). When compared between the heavier occlusal force side and the lighter occlusal force side, both exhibited a similar force distribution pattern. The side which had heavier occlusal force was significantly greater than the side with lighter occlusal force in both treatment approaches ( *p* < 0.001).

**Table 4:** Comparisons of the bilateral occlusal force balances between right and left sides and between heavy occlusal force side and light occlusal force side of postorthodontic treatment subjects with extraction and nonextraction approaches ( *n* = 18 for each group)

Treatment	Bite force (%)				*p* -Value	Bite force (%)				*p* -Value
	Right side		Left side			Heavy side		Light side		
	± SD	95% CI	± SD	95% CI		± SD	95% CI	± SD	95% CI	
Nonextraction	56.30 ± 12.46	50.10–62.49	42.80 ± 12.41	36.63–48.97	0.014 a	60.53 ± 8.69	56.21–64.85	38.56 ± 8.65	34.26–42.86	0.000 b
Extraction	55.41 ± 10.24	50.32–60.50	44.31 ± 10.10	39.28–49.33	0.094	57.70 ± 8.49	53.48–61.93	42.01 ± 8.35	37.86–46.17	0.000 b
*p* -Value	0.389		0.296			0.223		0.154		

Abbreviations: CI, confidence interval; SD, standard deviation.

a Statistically significant at *p* < 0.05.

b Statistically significant at *p* < 0.01 by Wilcoxon's signed ranks test for within-group comparison and Mann–Whitney's test for between-group comparison.

## Discussion

Our study aimed to examine the bite force distribution in postorthodontic subjects treated with extraction and nonextraction approaches. The two groups might reflect individuals with normal occlusion, having 24 teeth and 28 teeth. The extraction group consisted exclusively of patients who underwent the four-premolar extraction approach, allowing them to achieve a normal occlusion with a Class I molar relationship upon completing their orthodontic treatment. 16 26 Although there was a gender imbalance in our study, and previous research has shown that males have a greater total absolute biting force than females, 3 4 7 this did not affect our findings. This is because we measured bite force as a percentage.

The T-Scan III system used in this study measures the bite force distribution within the same arch as a percentage. Previous T-Scan studies have found that postorthodontic patients exhibit a higher bite force (82–89%) in the posterior region and a lower bite force (11–14%) in the anterior region compared with nonorthodontic patients (77–85 and 11–22%, respectively). 18 19 This study also found a similar bite force distribution pattern in postorthodontic patients treated with either nonextraction or extraction approaches. Additionally, the A-P ratio was measured to assess the force balance between anterior and posterior bite forces, which is important for ensuring optimal masticatory function and minimizing stress on the anterior teeth. Both the extraction and nonextraction groups in this study exhibited a similar A-P ratio to the postorthodontic group of Qadeer et al's study. 18 They also found the postorthodontic group had a lower A-P ratio than the nonorthodontic group (0.18 and 0.46, respectively). This could be because the normal occlusion achieved after orthodontic treatment distributes bite force primarily in the posterior regions during MIP to protect the anterior teeth and allow them to function properly. 16 17 Moreover, pretreatment malocclusions can result in unbalanced bite force distribution patterns, such as in patients with anterior open-bite occlusion or nonoccluded teeth. 27 The findings of this study demonstrate that both extraction and nonextraction treatment approaches can achieve a more balanced anteroposterior force distribution pattern.

In normal populations, individuals with fewer teeth have less bite force than people with more teeth. 3 8 9 12 13 14 However, in postorthodontic patients, the total bite force between nonextraction patients and extraction patients is not different. 28 29 Moreover, this study also found a similar pattern of bite force distribution in the anterior and posterior regions between the extraction and nonextraction groups. This finding suggests that after alignment and correction of tooth positions through orthodontic treatment, patients with 24 teeth may exhibit equivalent occlusal function in terms of bite force distribution compared with those with 28 teeth.

This study found that teeth of the same type exhibited similar bite force in both extraction and nonextraction approaches, except for the second molars, which had greater bite force in the extraction group. Previous T-Scan studies also found that postorthodontic treatment patients had high occlusal bite force (22–25%) and a high prevalence of dental interference in the second molars. 18 30 One reason for this could be that in the extraction group, the forward movement of the molars to close the extraction space may cause molar extrusion due to the orthodontic mechanism itself. 31 32 The second molars are the teeth prone to have more occlusal contacts than other teeth in the arch when the extrusion occurs during orthodontic space closure, leading to the increased bite force on the second molars in the extraction group. 33 Supporting this explanation, a study by Thabet et al (2023) using the T-Scan III system found significant force distribution imbalances and increased occlusal force on specific teeth, particularly the molars in orthodontic cases with premolar extraction compared with nonorthodontic normal occlusion subjects. 19

The symmetrical bilateral occlusal force distribution was considered as a healthy occlusal function which is predominantly found in the normal occlusion subjects. 18 19 34 However, the bilateral occlusal imbalance was found in this study and previous studies based on subjects in postorthodontic treatment. 18 19 35 They noted that this imbalance could be from the occlusal instability and premature contacts. 19 This finding suggested that the occlusal force balance evaluation and adjustment should be done prior or in the retention phase in the orthodontic patients.

The T-Scan system, with its computerized digital occlusal record, offers a more objective and consistent visualization of occlusal conditions in a consistent and repeatable manner. This can enhance treatment efficiency and patient outcomes. 36 Specifically in orthodontics, this tool can be valuable during the finishing process or in the completion of the treatment to check for a minor adjustment of the imbalanced occlusion, especially at the second molars, which could traumatize the bear teeth and lead to pathological conditions such as cervical lesions, periodontal problems, and patient discomfort. 19 35 36 37

Despite the similar distribution of bite force between the nonextraction and the four-premolar extraction orthodontic approaches found in this study, other possible consequences from the extraction treatment, such as the loss of healthy teeth, dish-in profile, temporomandibular disorders, anterior teeth inclination, and vertical facial dimension should be considered in the extraction treatment plan of each patient. 38

Based on this study, several aspects could be further analyzed or explored in future research, including the investigation of the relationship between pretreatment malocclusion types and bite force distribution. This study did not fully cover all types of malocclusions, particularly Class II malocclusions. Future research could include a more diverse sample of pretreatment malocclusions to understand better how they affect bite force distribution after the treatment. In addition, the inclusion of nonorthodontic patients with normal occlusion as a control group would allow for a comparison of bite force distribution patterns between patients with normal occlusion and postorthodontic patients, providing insights into the effects of orthodontic treatment on bite force distribution.

## Conclusion

In comparing the extraction and nonextraction approaches for orthodontic treatment, both exhibited similar bite force ratios between the anterior and posterior regions, with a greater amount of bite force in the posterior regions and less in the anterior areas. While the bite force distribution was consistent across most tooth types in both groups, the second molars in the extraction approach group exhibited significantly more force than those in the nonextraction group. Within the study's limitations, by recognizing the minor occlusal differences between the extraction and nonextraction treatment approach, the overall occlusal function from both approaches is expected to be comparable. However, more caution should be given to the bilateral occlusal balance, and second molars, in case we performed an extraction approach, whether they had any premature contacts or heavy occlusal force, which should be adjusted before or after the debonding process.
